# Research on robot path tracking method based on IDDPG-MPC

**DOI:** 10.1371/journal.pone.0350307

**Published:** 2026-07-06

**Authors:** Haicheng Shen, Xun Xu, Zhiqiang Miao

**Affiliations:** 1 School of Yonyou Digital and Intelligence, Nantong Institute of Technology, Nantong, Jiangsu, China; 2 Faculty of computing, Universiti Teknologi Malaysia, Johor Bahru, Johor, Malaysia; 3 Jiangsu Xinneng Haili Offshore Wind Power Generation Co.Ltd., Nantong, Jiangsu, China; Qingdao University, CHINA

## Abstract

In complex marine environments, path-following control of unmanned surface vessels (USVs) faces numerous challenges, including environmental disturbances, dynamic nonlinearities, and underactuated systems. To overcome the limitations of traditional line-of-sight/PID control in terms of robustness and adaptability, this study proposes a hybrid control architecture combining improved deep deterministic policy gradient (IDDPG) and model predictive control (MPC). The IDDPG algorithm, as the upper-level decision-making module, utilizes deep reinforcement learning to generate optimal heading angle increment commands by learning the environmental state. The MPC, as the lower-level execution module, optimizes control variables such as thrust and rudder angle through rolling optimization based on the USV’s three-degree-of-freedom nonlinear dynamics model. This study constructs a closed-loop “perception-decision-execution-learning” paradigm and employs gradient pruning and a customized reward function to ensure the stability of algorithm training and the optimality of control decisions. Lateral deviation and heading angle error are used as evaluation metrics to verify the control performance. Simulation results show that this method effectively solves the adaptability challenge of traditional control strategies in complex environments. Compared with the traditional ALOS-PID method, the average lateral deviation is reduced by 37% and the heading angle error is reduced by 21%, thus realizing high-precision path tracking control for unmanned surface vessels and providing a new method for autonomous surface vehicle navigation.

## 1. Introduction

The rapid development of marine intelligent equipment technology has made unmanned surface vessels (USVs) core equipment for tasks such as marine exploration, environmental monitoring, and maritime patrol [1]. As a typical underactuated nonlinear system, USVs are only equipped with sway thrust and yaw control torque, lacking direct lateral force control [2, 3]. Under strong and uncertain ocean disturbances such as wind, waves, and currents, their high-precision path tracking control faces severe challenges. Path tracking requires USVs to navigate strictly along a preset reference trajectory based on environmental perception and their own motion state, while simultaneously meeting multiple constraints such as motion stability, real-time control, and strong anti-interference capabilities. It is the core technology for autonomous navigation of USVs. Traditional path tracking methods, represented by line-of-sight (LOS) and PID control, are widely used due to their simple structure and ease of implementation [4–7]. However, these methods have significant drawbacks in complex sea conditions: poor robustness, making it difficult to suppress trajectory deviations caused by continuous environmental disturbances; weak nonlinear adaptability, failing to match the highly nonlinear dynamic characteristics of USVs; and insufficient anti-interference capability, easily leading to overshoot and tracking failure under model mismatch and sudden disturbances, making it difficult to meet the requirements of high-precision and high-reliability operations [8–11].

In recent years, reinforcement learning and model predictive control (MPC) have provided new ideas for solving these problems. Deep Deterministic Policy Gradient (DDPG) is a deep reinforcement learning algorithm with adaptive environment learning capabilities. It can autonomously optimize policies through interaction with the environment, effectively handling system uncertainties and external disturbances, and can directly output continuous heading control commands, making it suitable for USV high-dimensional continuous action space decision-making [12–14]. However, a single DDPG has problems such as limited real-time control capabilities, difficulty in meeting the physical constraints of actuators, and easy training divergence. Model predictive control (MPC), relying on rolling optimization and feedback correction mechanisms, can explicitly handle system constraints, achieve precise low-level control based on predictive models, and has excellent stability and anti-interference execution capabilities [15–17]. However, MPC relies heavily on accurate dynamic models and lacks adaptability in scenarios involving sudden environmental changes and model mismatch, leading to a tendency for tracking errors to accumulate [18].

Therefore, DDPG and MPC exhibit strong complementarity in performance: DDPG excels in adaptive decision-making and disturbance rejection learning, while MPC excels in constraint handling and precise execution. Organically integrating the two can construct a hybrid control architecture that combines environmental adaptability and precise execution, overcoming the shortcomings of a single algorithm [19–21]. To this end, this paper proposes an improved deep deterministic policy gradient-model predictive control (IDDPG-MPC) hierarchical hybrid path tracking method, addressing the high-precision tracking requirements of underactuated USVs under wind, wave, and current disturbances, and constructing a closed-loop control architecture of “perception-decision-execution-learning.” The upper layer employs the IDDPG algorithm, improving policy convergence and environmental adaptability through gradient pruning, customized composite reward functions, and state-space optimization, outputting the optimal heading angle increment command; the lower layer employs MPC, performing roll optimization based on the USV’s three-degree-of-freedom nonlinear dynamic model to generate thrust and rudder angle control quantities, achieving precise execution. By employing a two-layer collaborative coupling approach, balancing decision optimization and local real-time control, the proposed method significantly improves tracking accuracy, robustness, and anti-interference capability. Simulation results demonstrate that, compared to the traditional ALOS-PID method, the proposed method effectively reduces lateral deviation and heading angle error, exhibiting superior tracking performance in both straight-line and piecewise linear tracking scenarios. This provides a feasible solution for autonomous path tracking of underactuated USVs in complex marine environments.

## 2. Theoretical foundation and model construction

### 2.1. Theoretical basis

#### 2.1.1. Deterministic strategy theory.

DDPG is a deep reinforcement learning algorithm for continuous action space, proposed by Lillicrap et al. in 2016. It combines the advantages of deep Q network (DQN) and policy gradient method, and is suitable for tasks that require high-precision continuous action output, such as robot control and autonomous driving. The core idea of DDPG comes from the deterministic policy gradient theorem, which proves that under a deterministic strategy (i.e., given a state, output a certain action), the policy gradient can still be effectively optimized through the gradient of the value function.

DDPG adopts the Actor-Critic architecture, in which the Actor network (policy network) is responsible for outputting deterministic actions, namely:


$$\begin{array}{c} a_{t}=\mu \left(s_{t}|\theta^{\mu }\right) \end{array}$$ (1)

Among them, μ (⋅) is the policy function, and $\theta^{\mu }$ is the parameter of the Actor network. The Critic network (Q network) is used to evaluate the value of the state-action pair, and its output is:


$$\begin{array}{c} Q(s_{t},a_{t}|\theta^{Q}) \end{array}$$ (2)

The optimization goal of the Critic network is to minimize the temporal difference (TD) error:


$$\begin{array}{c} L\left(\theta^{Q}\right)=E_{\left(s_{t},a_{t},r_{t},s_{t+\text{1}}\right)\sim D}\left[{\left(Q\left(s_{t},a_{t}|\theta^{Q}\right)-y_{t}\right)}^{\text{2}}\right] \end{array}$$ (3)

Among them, the target value $y_{t}$ is calculated by the target network:


$$\begin{array}{c} y_{t}=r_{t}+\gamma Q^{\prime }\left(s_{t+\text{1}},\mu^{\prime }\left(s_{t+\text{1}}|\theta^{\mu^{\prime }}\right)|\theta^{Q^{\prime }}\right) \end{array}$$ (4)

Here, γ is the discount factor (0<γ<1), $\mu^{\prime }$and $Q^{\prime }$ are the target Actor and target Critic network respectively, and its parameters are slowly updated through soft update: this mechanism helps to stabilize the training process and avoid drastic fluctuations in Q-value estimation.


$$\begin{array}{c} \theta^{Q^{\prime }}\leftarrow \tau \theta^{Q}+(\text{1}-\tau )\theta^{Q^{\prime }} \end{array}$$ (5)

$$\begin{array}{c} \theta^{\mu^{\prime }}\leftarrow \tau \theta^{\mu }+(\text{1}-\tau )\theta^{\mu^{\prime }} \end{array}$$ (6)

Among them, τ is the soft update rate, and its value is a small positive number (generally 10^3^ ~ 10^4^).

Traditional DDPG algorithms may face problems such as slow policy convergence, gradient explosion, and insufficient robustness in complex and dynamic marine environments. The IDDPG algorithm proposed in this study mainly incorporates three improvements over the traditional DDPG algorithm: 1. Gradient pruning technique: Calculating the L2 norm of the gradient vector during network parameter updates and scaling excessively large gradients to prevent gradient explosion and ensure training stability; 2. Customized reward function design: Constructing a multi-objective composite reward function that includes lateral tracking error, heading angle error, and control force to achieve synergistic optimization of tracking accuracy and control smoothness; 3. State space optimization: Selecting key state variables (position, velocity, heading angle, tracking error) related to USV path tracking as network inputs to reduce the dimensionality of the state space and improve the algorithm’s learning efficiency. Both DDPG and IDDPG employ an experience replay mechanism, storing the agent’s experience of interacting with the environment in a replay pool and training on randomly selected batches of data. This not only helps improve training efficiency but also breaks down the correlation between data. The pseudocode of the IDDPG algorithm is shown in Table 1.

**Table 1:** DDPG pseudocode.

Algorithm 1 DDPG pseudo code
**1: Input:** Initial Actor network parameters, Critic network parameters, target Actor network parameters, target Critic network parameters, experience revisit pool D, discount factor, learning rate a, soft update rate τ
**2:** Initialization: Actor and Critic networks, target network $\theta^{\prime }$ = θ, $w^{\prime }$ =w
**3: for** each training cycle **do**
**4:** Initialization state *s*_*0*_
**5: for at** each time step *t = 0,1,...,T − 1* **do**
**6:** Selecting actions from an Actor network
**7:** Execute action at and get reward rt and next state st + 1 **8:** Store the transfer st, at, rt, st + 1 into the experience replay pool D **9:** Randomly sample mini-batches from the replay pool D **10:** Calculate the target Q value of Critic: ${\hat{Q}}_{t}=r_{t}+\gamma \cdot Q^{\prime }\left(s_{t+\text{1}},a_{t+\text{1}}\right)$
**11:** Update Critic Network: Minimize Loss Function $L_{C}=\frac{\text{1}}{N}\sum_{i=\text{1}}^{N}{\left(Q\left(s_{i},a_{i}\right)-{\hat{Q}}_{t}\right)}^{\text{2}}$
**12:** Calculate the gradient of the Actor: $\nabla_{\theta }L_{A}=\frac{\text{1}}{N}\sum_{i=\text{1}}^{N}\nabla_{\theta }\text{log}\pi_{\theta }\left(a_{i}|s_{i}\right)\cdot Q\left(s_{i},a_{i}\right)$
**13:** Update the Actor Network: $\theta^{\mu }=\theta^{\mu }-\alpha \cdot \nabla_{\theta^{\mu }}L_{A}$
**14:** Soft update target network: $\theta^{Q^{\prime }}\leftarrow \tau \theta^{Q}+(\text{1}-\tau )\theta^{Q^{\prime }}$ $\theta^{\mu^{\prime }}\leftarrow \tau \theta^{\mu }+(\text{1}-\tau )\theta^{\mu^{\prime }}$
**15:** **end for**
**16: end for**

The IDDPG algorithm process utilizes value-based Critic and policy-based Actor, and ensures efficient learning of reinforcement learning tasks in high-dimensional, continuous action space through key technologies such as experience replay, soft update and gradient clipping, which is more suitable for the complex and dynamic USV path tracking task.

#### 2.1.2. Model predictive control.

MPC is an advanced control strategy based on rolling optimization and feedback correction. It is widely used in fields such as robot control and process industry that require high-precision dynamic adjustment. Its core idea is to solve an optimal control problem in a finite time domain based on the current system state and the prediction model in each control cycle, and only execute the first control quantity and then re-predict and optimize in the next sampling cycle. This rolling optimization mechanism enables MPC to effectively handle system constraints and has strong robustness to model errors and external interference.

The mathematical description of MPC usually contains three key components, namely the prediction model, the objective function and the constraints. The prediction model is used to describe the dynamic behavior of the system. It can be a state space model, a transfer function or a data-driven black box model. Take the discrete state space model as an example:


$$\begin{array}{c} x_{k+\text{1}}=Ax_{k}+Bu_{k} \end{array}$$ (7)

$$\begin{array}{c} y_{k}=Cx_{k} \end{array}$$ (8)

Among them, $x_{k}$ is the system state, $u_{k}$ is the control input, and $y_{k}$ is the output. MPC solves the following optimization problem at each time k:


$$\begin{array}{c} \underset{u_{k|k},\ldots ,u_{k+N-\text{1}|k}}{min}\;\sum_{i=\text{0}}^{N-\text{1}}\;\left(∥x_{k+i|k}-x_{ref}\overset{\text{2}}{\underset{Q}{∥}}+∥u_{k+i|k}\overset{\text{2}}{\underset{R}{∥}}\right)+∥x_{k+N|k}-x_{ref}\overset{\text{2}}{\underset{P}{∥}} \end{array}$$ (9)

Among them, N is the prediction time domain, Q and R are the weight matrices of state and input respectively, and P is the terminal cost weight. The optimization problem also needs to meet the system constraints:


$$\begin{array}{c} u_{min}\leq u_{k+i|k}\leq u_{max} \end{array}$$ (10)

$$\begin{array}{c} x_{min}\leq x_{k+i|k}\leq x_{max} \end{array}$$ (11)

Among them, $u_{min}$, $u_{max}$ are the upper and lower limits of the control input, and $x_{min}$, $x_{max}$ are the upper and lower limits of the system state.

For the nonlinear dynamic system of USVs, this study adopts the nonlinear model predictive control (NMPC) method, which uses the 3-DOF nonlinear dynamic model of USVs as the prediction model, and solves the nonlinear optimization problem in each control cycle to generate the optimal control quantity, which is more in line with the actual motion characteristics of USVs.

### 2.2. USV Dynamic and kinematic model construction

This study focuses on USVs (Unmanned Surface Vessels) where only pitch thrust and yaw moment are controllable, with no active lateral thrust and passive lateral motion. A dual-coordinate system motion model is established, incorporating both the NED world coordinate system and the USV body coordinate system, with position, velocity, and heading angle as the core state variables. A three-degree-of-freedom nonlinear dynamic model (covering yaw, roll, and roll motions) is employed to describe the USV’s motion characteristics. This model achieves a balance between accuracy and computational complexity, making it highly suitable for meeting the real-time control requirements of USV path tracking.

The robot is a typical nonlinear underactuated system, and its motion model is described by a three-degree-of-freedom model, including lateral, longitudinal motion and yaw angle. The dynamic equation of the robot can be expressed as shown in formula (12):


$$\begin{array}{c} \text{M}\dot{\nu }+\text{C}(\nu )\nu +\text{D}(\nu )\nu =\tau \end{array}$$ (12)

Among them, M is the inertia matrix, C(v) is the Coriolis force and centrifugal force matrix, D(v) is the damping matrix, ${\nu =[u,v,r]}^{T}$ represents the velocity vector, which includes the longitudinal velocity u, the lateral velocity v and the yaw angular velocity r, ${\tau =[\tau_{u},\tau_{v},\tau_{r}]}^{T}$ is the control input, the longitudinal thrust is *tauu*, the lateral thrust is *tauv* and the yaw moment is *taur*. The specific calculation of each matrix is as follows:


$$\begin{array}{c} M=[\begin{array}{ccc} m-X_{u} & \text{0} & \text{0}\\ \text{0} & m-Y_{v} & m_{xg}-Y_{r}\\ \text{0} & m_{xg}-Y_{r} & I_{z}-N_{r} \end{array}] \end{array}$$ (13)

$$\begin{array}{c} C(v)=[\begin{array}{ccc} \text{0} & \text{0} & -\left(m-Y_{v}\right)v-\left(m_{xg}-Y_{r}\right)r\\ \text{0} & \text{0} & \left(m-X_{u}\right)u\\ \left(m-Y_{v}\right)v+\left(m_{xg}-Y_{r}\right)r & -\left(m-X_{u}\right)u & \text{0} \end{array}] \end{array}$$ (14)

$$\begin{array}{c} D(v)=[\begin{array}{ccc} -X_{u} & \text{0} & \text{0}\\ \text{0} & -Y_{v} & -Y_{r}\\ \text{0} & -N_{v} & -N_{r} \end{array}] \end{array}$$ (15)

Each element in the matrix (such as $X_{u}$、$Y_{v}$, etc.) is a hydrodynamic coefficient, which represents the fluid dynamic characteristics of the robot in different states. The above equations describe the robot’s movement in water, the longitudinal, lateral and yaw dynamic behavior. Considering the under-actuated characteristics of the robot, the control input is mainly generated by the thrust T and thrust angle δ of the propeller, as shown in formula (16): where $x_{l}$ represents the distance between the point of action of the propeller and the center of mass of the robot.


$$\begin{array}{c} \left\{ \begin{array}{c} @l\tau_{u}=T\text{cos}(\delta )\\ \tau_{v}=T\text{sin}(\delta )\\ \tau_{r}=x_{l}T\text{sin}(\delta ) \end{array}\right.\; \end{array}$$ (16)

$$\begin{array}{c} \dot{\eta }=\text{R}(\psi )\nu \end{array}$$ (17)

The robot’s kinematic model describes the motion in the geographic coordinate system in detail, and its form is as shown above. In order to represent the robot’s position information, including the lateral position *x*, longitudinal position *y* and heading angle *ψ, R(ψ)* is the heading angle rotation matrix, so the velocity is converted from the hull coordinate system to the geographic coordinate system. In order to ensure the stability and accuracy of subsequent control, the key parameters in the dynamic model are listed, and each variable is defined and explained. The definitions of the robot symbols are shown in Table 2.

**Table 2:** Definitions of robot symbols.

Symbol	Definition
x, y, z	The robot’s position in the world coordinate system (vertical, horizontal, vertical)
φ, θ, ψ	Euler angles of the robot (roll, pitch, heading)
u, v, r	Velocity vector (longitudinal velocity, lateral velocity, yaw rate)
M	Inertia Matrix
C(v)	Coriolis and Centrifugal Force Matrix
D(v)	Damping Matrix
$\tau_{u},\tau_{v},\tau_{r}$	Control input (longitudinal thrust, lateral thrust, yaw moment)
$x_{l}$	The distance between the propeller action point and the center of mass of the unmanned boat
R(ψ)	Heading angle rotation matrix

### 2.3. Mathematical modeling of IDDPG-MPC hybrid control

#### 2.3.1. Model predictive control.

**Fig 1:**
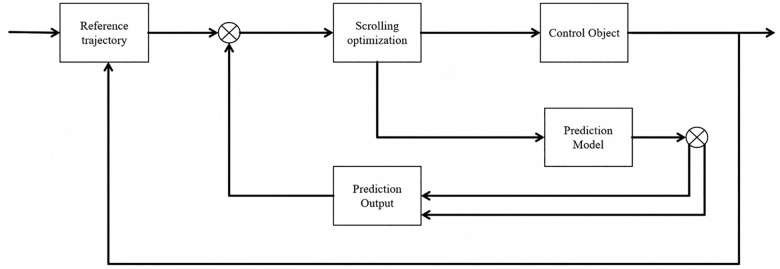
Basic framework of MPC control.

Model predictive control is an advanced control strategy with optimization as the goal. It is a commonly used advanced control system for many advanced control projects. The algorithm makes the best estimate of the state of a period of time in the future based on the mathematical model of the system at each sampling moment, and then constructs and solves a corresponding problem with the predicted value of this time period, and obtains the optimal solution as the control quantity for the current moment to input to the system. This includes the process of making judgments about future developments, and has good robustness when facing some unknown or highly variable systems and environments. In addition to being able to consider various constraints at the same time, the most prominent advantage of MPC is that it uses a rolling optimization method. Its feedback loop has a certain degree of predictability, and can still achieve good control effects when facing adverse factors such as external interference and parameter uncertainty. Therefore, for some complex nonlinear systems with multiple variables and different constraints, we can use MPC to complete related work. The core idea of MPC is to achieve robust control of dynamic systems through rolling time domain optimization and feedback correction. The mathematical forms of linear and nonlinear MPC are derived in detail, and the engineering challenges of real-time solution are discussed. Fig 1 is a flow chart of the entire MPC method.

MPC achieves precise control in dynamic environments through a closed-loop mechanism of prediction-optimization-execution. The algorithm first predicts the state evolution trajectory in the future based on the current state and dynamic model of the system, and then solves the finite time domain optimization problem to calculate the optimal control sequence that minimizes the objective function. The objective function usually includes trajectory tracking error, control input energy and constraint violation penalty term, and its mathematical expression is a constrained quadratic programming problem. MPC only executes the first control quantity of the optimal sequence and re-predicts and optimizes in the next sampling period to form a rolling horizon control strategy. This mechanism gives MPC three core advantages: first, disturbance feedforward compensation is achieved through model prediction; second, physical limitations such as input saturation and state constraints are explicitly handled; and third, adaptation to time-varying environments is achieved through periodic optimization. In the path tracking application of underwater robots, MPC can effectively handle motion constraints such as speed and heading angle. Compared with the hysteresis compensation of traditional PID and the instability risk of reinforcement learning, MPC combines the accuracy of model prediction and the stability of optimization control. By combining DDPG’s path tracking with MPC’s local dynamic optimization, the rationality of long-term strategies is ensured, while the accuracy of short-term control is achieved, forming complementary advantages. This hybrid architecture gives full play to MPC’s ability to handle system constraints and reinforcement learning’s ability to adapt to the environment, providing a reliable solution for robot control in complex marine environments.

#### 2.3.2. Markov decision process.

As the core mathematical model of reinforcement learning, Markov decision process represents how robots take actions in reinforcement learning. With the knowledge and understanding of decision-making methods and their expressions, robots can make decisions in different states, act based on their own situations, and gradually become more long-term based on the effects of their decisions. In the Markov decision process, there are ways in which the environment interacts with the robot and the events that occur. With the help of this knowledge, the robot can find the right decision and implement it, so that it can achieve the task of accumulating the most rewards.

The reinforcement learning process follows the basic paradigm of “perception-decision-execution-learning”. The robot first perceives the current state of the environment and selects and executes the corresponding action based on the established strategy; the environment then generates state transitions based on the robot’s actions and gives immediate reward feedback; the robot continuously adjusts its own strategy to optimize long-term performance by analyzing the new state and the rewards obtained. This cyclic mechanism enables the robot to gradually improve its decision-making ability through repeated attempts and finally achieve the expected goal. The whole process is based on the theory of Markov decision process. Its core feature is that the decision at each moment depends only on the current state and is independent of the historical state. This property greatly simplifies the complexity of the decision-making process. The Bellman equation plays a key role in the theoretical system of reinforcement learning. It decomposes complex long-term decision-making problems into a combination of current immediate rewards and subsequent state values, thereby establishing a value assessment system that runs through the entire decision-making process. For practical applications such as robot path tracking, this theoretical framework is concretized as follows: the robot obtains its own position, heading and other state information through sensors, the control system generates propulsion instructions and other actions according to the current state, and the environment returns reward signals containing path deviation, energy consumption and other indicators. By continuously accumulating these interactive experiences, the robot gradually learns to make optimal decisions in complex environments, such as avoiding obstacles while maintaining track accuracy. This autonomous learning method based on actual feedback enables the robot to adapt to dynamically changing working environments, showing flexibility and robustness that traditional programmed control methods do not have.

**Fig 2:**
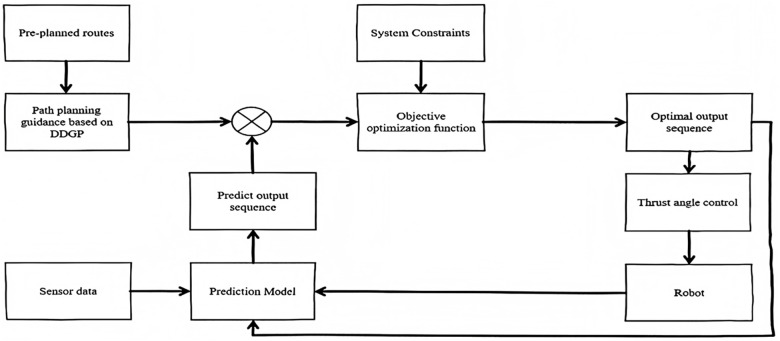
Reinforcement learning controller.

The autonomous navigation control framework based on deep reinforcement learning and model predictive control collaborative optimization realizes path tracking in dynamic environments through a hierarchical decision-making mechanism. In the framework of Fig 2, the DDPG algorithm is used as a high-level decision-making unit to analyze the heading angle setting value of the reference path from a global perspective and generate strategy instructions that meet the multi-objective optimization requirements, while the MPC is used as a low-level execution unit to solve the optimal control sequence in a limited time domain according to the real-time environmental perception data and dynamic constraints, and finally output thrust distribution optimization instructions. The controller realizes sensor data fusion and motion state estimation through the observer and combines the forward prediction model to build a dynamic feedback regulation loop. The core innovation of this framework lies in the integration of the autonomous strategy learning ability of deep reinforcement learning and the constraint processing ability of model predictive control. First, the DDPG module obtains environmental adaptability strategy parameters through offline training to effectively deal with unstructured interference. Second, the MPC module ensures that the control instructions meet the dynamic constraints and real-time safety boundaries of the propulsion system through online rolling optimization. The synergy of the two is reflected in three levels. First, the high-level strategy output by DDPG provides MPC with a dynamically adjusted reference trajectory to alleviate the accumulation of tracking errors caused by the mismatch of the prediction model in traditional MPC. Second, the real-time optimization results of MPC provide local environmental feedback for the DDPG policy network to enhance the online adaptability of the intelligent agent to sudden disturbances. Third, the dual modules realize information interaction through the shared state feature space, and build a dynamic balance mechanism between strategy exploration and constraint satisfaction.

Based on the mathematical framework of Markov decision process, this study constructs a deep reinforcement learning-model predictive control (DRL-MPC) hybrid system for robot motion control. By designing a composite reward function to drive the gradient update of the policy network, the rolling time domain optimization characteristics of MPC are used to make feasibility corrections to the action instructions generated by DDPG.

## 3. USV path tracking algorithm based on IDDPG-MPC

### 3.1. Algorithm principle

**Fig 3:**
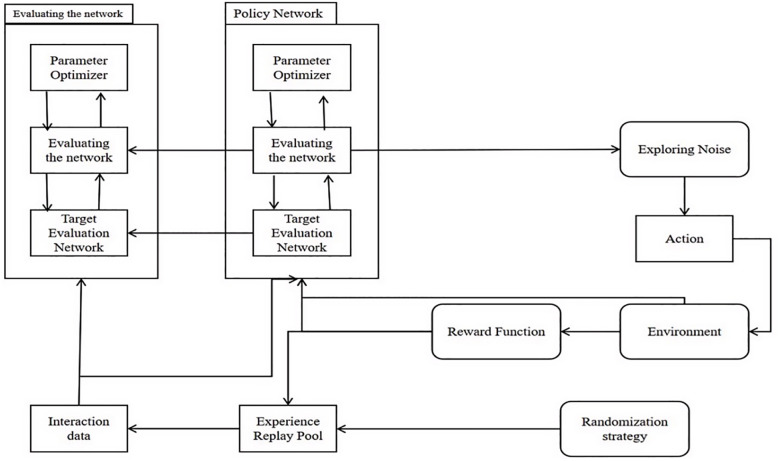
Algorithm framework diagram.

To address the dual requirements of high accuracy and robustness for path tracking tasks of USVs in complex marine environments, this study proposes a hierarchical collaborative optimization architecture that integrates an improved IDDPG and MPC. This architecture decomposes the USV path tracking task into two parts: high-level heading angle decision-making and low-level motion control execution, effectively combining the environmental adaptability of IDDPG with the real-time optimization and constraint handling characteristics of MPC. The overall algorithm framework is shown in Fig 3.

The path tracking control scheme used in this study is mainly composed of two core modules: the policy network (Actor) and the evaluation network (Critic), and intelligent decision-making is achieved through interactive learning. In the policy network part, it contains a dual structure of online policy network and target policy network. The online network receives the state $S_{t}$ and outputs action suggestions, while introducing exploration noise to form the final action to promote exploration; the target network slowly tracks the parameters of the online network through the soft update mechanism $\theta^{\prime }\leftarrow \tau \theta +(\text{1}-\tau )\theta^{\prime }$ to ensure training stability. The evaluation network part adopts a similar twin architecture. The online evaluation network is used to calculate the state-action value function, and the target evaluation network keeps the parameters synchronized through soft update $w^{\prime }\leftarrow \tau w+(\text{1}-\tau )w^{\prime }$. The two together constitute a dual approximator of the Bellman equation. When the system is running, after the agent applies the action generated by the policy network to the environment, it collects the transfer samples containing the new state and immediate reward and stores them in the experience replay pool, and performs batch training by breaking the data correlation through random sampling. During the parameter update process, the evaluation network uses the mean square Bellman error minimization criterion to update the value function parameters, and the policy network adjusts the policy parameters along the direction of the value function gradient ascent, forming a virtuous cycle of “evaluation-improvement”. It is particularly noteworthy that the framework has set up independent parameter optimizer modules, which are responsible for the gradient update calculation of the policy network and the evaluation network respectively, and the gradient back propagation path is clearly marked by the dotted arrow. The entire architecture effectively solves the problems of data correlation, target fluctuation and insufficient exploration in reinforcement learning through key technologies such as experience replay, target network and exploration noise, and finally achieves continuous optimization of the strategy.

In the top-level design, the DDPG algorithm, as a global path planner, implements intelligent decision-making through the Actor-Critic architecture constructed by deep neural networks. The algorithm uses the experience replay mechanism and target network technology to ensure stable learning in complex environments. Specifically, the Actor is responsible for mapping the robot’s multi-dimensional state observations into heading angle commands in the continuous action space, while the Critic evaluates the long-term benefits of the action and guides the policy optimization through the time difference error. This design enables DDPG to gradually adapt to environmental changes and provide the system with a forward-looking heading reference. At the bottom control level, the MPC module, as a local optimizer, uses the heading angle output by DDPG as the reference trajectory combined with an accurate dynamic model and real-time sensor data to solve the optimal control problem in the rolling time domain framework. The MPC controller uses a numerical optimization algorithm to calculate the propulsion force and rudder angle commands in real time by constructing a multi-objective optimization function that includes tracking error, control energy consumption, and actuator constraints. This design not only ensures the accuracy of control but also effectively handles various physical constraints. The two modules are tightly coupled through data flow: DDPG continuously optimizes the long-term strategy based on environmental feedback to provide MPC with a dynamically adjusted reference path; MPC helps DDPG better understand the dynamic characteristics of the environment through real-time control feedback to form a virtuous cycle. The innovation of this collaborative architecture is reflected in three aspects: first, it breaks through the limitations of a single algorithm. DDPG solves the environmental adaptability problem of MPC in long-term planning, and MPC makes up for the shortcomings of DDPG in instantaneous control; second, through hierarchical design, decoupling on the time scale is achieved. DDPG focuses on the slow-changing global strategy and MPC handles the fast-changing local optimization; finally, the two-way interaction of data flow forms a closed-loop learning mechanism that enables the system to have the ability to continuously improve. Experimental results show that this architecture enables the robot to achieve accurate path tracking in a complex dynamic environment, and respond quickly to local disturbances while maintaining global navigation performance, showing adaptability and robustness that are difficult to achieve with traditional control methods. Among them, $Actor(S_{t})$ is a strategy implemented by a deep neural network to generate the desired heading angle $\varphi_{d}$.


$$\begin{array}{c} \varphi_{d}=Actor(S_{t}) \end{array}$$ (18)

For the rolling optimization mechanism, MPC uses the target heading angle $\varphi_{d}$, combined with the current state $S_{t}$, to calculate the optimal control input (thrust and rudder angle). The optimization target of MPC is as follows. In the formula, $\varepsilon_{i}$ is the path tracking error (i.e., the deviation between the desired heading angle and the current heading angle). $\Delta \psi_{i}$ is the change in the rudder angle. $u_{i}$ is the thrust. *Q*, *R* and *K* are weight coefficients to balance the control error with the input change.


$$\begin{array}{c} \underset{\Delta \psi ,u}{\text{min}}\sum_{i=\text{1}}^{N}\left(Q\cdot \overset{\text{2}}{\underset{i}{\epsilon }}+R\cdot \Delta \overset{\text{2}}{\underset{i}{\psi }}+K\cdot \overset{\text{2}}{\underset{i}{u}}\right) \end{array}$$ (19)

The purpose of this formula is to optimize the robot’s rudder angle and thrust input by minimizing errors and control input changes so that the robot can accurately drive along the predetermined path. After executing the control, the robot’s state information can be fed back to the DDPG and MPC modules through sensors. DDPG optimizes the global path guidance strategy $\psi_{d}$ based on the new state *S*_t+1_, and MPC re-optimizes the control inputs Δψ and *u* for the new state. The robot dynamics model in the formula is used to predict the new state $S_{\text{t}+\text{1}}$. Under closed-loop control, DDPG and MPC continuously adjust the control strategy for real-time feedback to ensure high accuracy and high robustness of path tracking.


$$\begin{array}{c} S_{t+\text{1}}=f\left(S_{t},\Delta \psi ,u\right) \end{array}$$ (20)

### 3.2. Algorithm model

This study proposes a mathematical model of the steering gear-yaw system of a USV based on the IDDPG-MPC collaborative control framework. The model adopts a hierarchical architecture design, organically combines intelligent decision-making with precise control, and provides a complete theoretical framework for the path tracking control of USV. In terms of system dynamics modeling, a state equation with the heading angle *ψ* and the yaw rate *r* as the core is established. Heading dynamics equation:


$$\begin{array}{c} \dot{\psi }(t)=r(t) \end{array}$$ (21)

It describes the change of heading angle over time, while the position update equations $\dot{x}(t)=v\text{cos}(\psi (t))$ and $\dot{y}(t)=v\text{sin }(\psi (t))$ establish the mapping relationship between heading and position change, where v is the constant ship speed. This set of equations fully describes the motion characteristics of the USV on the horizontal plane and lays a theoretical foundation for the subsequent control algorithm design.

The core innovation of the control system lies in the realization of the hierarchical design concept. The high-level control uses the DDPG algorithm to generate the reference heading increment $\Delta \psi_{d}$ through the Actor network. The network parameter $\theta^{\mu }$ is continuously optimized through reinforcement learning to enable the system to have environmental adaptability. The low-level control is based on the MPC framework, using the first-order steering gear model


$$\begin{array}{c} T\dot{r}(t)+r(t)=K\delta (t) \end{array}$$ (22)

Describe the dynamic characteristics of the steering gear system, where *T* is the time constant, *K* is the gain coefficient, and δ(t) is the rudder angle command output by the MPC. This hierarchical structure ensures both the intelligence of path tracking and the accuracy of local control. The error definition link introduces two key performance indicators, namely lateral error ε(t) and heading error β(t). Lateral error: quantifies the deviation between the actual position of the USV and the expected path;


$$\begin{array}{c} \varepsilon (t)=-\left(x(t)-x_{\text{0}}\right)\text{sin }\psi_{p}+\left(y(t)-y_{\text{0}}\right)\text{cos }\psi_{p} \end{array}$$ (23)

Heading error: The difference after periodic processing is used to characterize the heading control accuracy. These two error indicators provide clear optimization targets for the control algorithm.


$$\begin{array}{c} \beta (t)=\text{wrap}\left(\psi_{d}(t)-\psi (t)\right)=\left[\psi_{d}(t)-\psi (t)+\pi \right]\text{mod2}\pi -\pi \end{array}$$ (24)

To ensure the stability of deep neural network training, the model uses gradient clipping technology. This technology calculates the L-2 norm $∥\text{g}{∥}_{\text{2}}$of the gradient vector g and compares it with the preset threshold c to scale the excessive gradient. The specific operation is defined as: when $∥\text{g}{∥}_{\text{2}}$≤c, keep the original gradient, and when $∥\text{g}{∥}_{\text{2}}$>c, scale the gradient to $\frac{c}{∥\text{g}{∥}_{\text{2}}}\text{g}$. This mechanism effectively prevents the gradient explosion problem during training and improves the convergence and stability of the algorithm.

### 3.3. Algorithm flow

The IDDPG-MPC collaborative control algorithm in this article adopts a hierarchical and progressive execution architecture, and realizes intelligent path tracking through the organic combination of global planning and local optimization. Three key preparations need to be completed in the algorithm initialization phase: first, conFig the dual structure of the DDPG network, including the Actor responsible for action generation and the Critic for evaluating the value of the action; second, set the optimization objective function and related weight parameters of the MPC controller; finally, load the robot dynamics model as the prediction basis. In the formal operation phase, the system continuously optimizes in an iterative manner: at the beginning of each control cycle, the algorithm obtains the current state information of the robot, such as posture and speed, and synchronously updates the preset target path data. The DDPG module calculates the expected heading angle command in real time through the policy network based on the deep reinforcement learning mechanism, and stores the state transition samples generated by the environment interaction into the experience replay pool and uses the randomly sampled historical data to iteratively update the network parameters. This process enables the system to have the ability of continuous learning and environmental adaptation.

As the execution layer of the algorithm, the MPC control module receives the heading angle reference command output by DDPG and performs multi-objective rolling optimization in combination with real-time sensor data. This module first calculates the deviation between the current state of the robot and the target trajectory, and then solves the optimal control sequence in the future finite time domain through numerical optimization under the premise of considering the dynamic constraints, and immediately executes the first control quantity to achieve rapid response. The entire control process forms a closed-loop feedback: the execution effect of MPC is fed back to the DDPG module through new state observations, prompting the policy network to continuously adjust the output instructions; at the same time, DDPG provides a more reasonable reference trajectory for MPC through continuous learning optimization strategies. This two-way data interaction mechanism enables the system to have both the intelligence of long-term planning and the accuracy of short-term control, which can adapt to environmental changes and meet real-time requirements. It is particularly noteworthy that the algorithm adopts a modular design concept. The two core modules maintain independent functions and work together through standardized interfaces, which not only facilitates system debugging and maintenance, but also reserves space for subsequent functional expansion. The path tracking pseudo code is shown in Table 3.

**Table 3:** Path tracking control pseudo code.

Algorithm 2 Pseudocode of path tracking control based on DDPG and MPC
Initialize the Actor network and the Critic network Initialize target networks $\mu^{\prime }{\text{ and }Q}^{\prime }$，weight$\theta^{\mu^{\prime }}\leftarrow \theta^{\mu }$，$\theta^{Q^{\prime }}\leftarrow \theta^{Q}$ Initialize the experience replay bufferD for each episode = 1 … M do state ← Env.reset() for t = 1 … T do 1) Actor selects the high level heading increment $a\leftarrow \mu \;(state|\theta^{\mu })+N(\text{0},\;\sigma )$ # 2) Environment stepping: MPC calculates low-level yaw rate and updates USVModel next_state, reward ← Env.step(a) # 3) Storage transfer (state, action, reward, next_state) 𝓓.add(state, a, reward, next_state) # 4) If the experience pool size≥ batch_size，update the network if |D| ≥ batch_size then # Randomly sample a batch ($s_{i},a_{i},r_{i},{s_{i}}^{\prime })$←𝓓 𝓓.sample(batch_size) # Critic update: Minimize the mean square error between Q($s_{i},a_{i}$) and the TD target $y_{i}$=$r_{i}+\gamma$·$Q^{\prime }$(${s_{i}}^{\prime },\;\mu^{\prime }({s_{i}}^{\prime }|\theta^{\mu^{\prime }})|\theta^{Q^{\prime }})$ $L=(\text{1}/N)\sum (Q(s_{i},a_{i}|\theta^{Q})-y_{i}{)}^{\text{2}}$ $\theta^{Q}\leftarrow \theta^{Q}-\eta_{Q}\nabla_{\theta^{Q}}L$ clip_grad_norm($\theta^{Q},c$) #Actor Update: Maximize Q value via gradient ascent $J=(\text{1}/N)\sum Q(s_{i},\mu (s_{i}|\theta^{\mu })|\theta^{Q})$ $\theta^{\mu }\leftarrow \theta^{\mu }+\eta_{\mu }\nabla_{\theta^{\mu }}J$ clip_grad_norm($\theta^{\mu },c$) # Soft update target network parameters $\theta^{Q^{\prime }}\leftarrow \tau \theta^{Q}+(\text{1}-\tau )\theta^{Q^{\prime }}$ $\theta^{\mu^{\prime }}\leftarrow \tau \theta^{\mu }+(\text{1}-\tau )\theta^{\mu^{\prime }}$ end if state ← next_state end for end for

Based on the above algorithm ideas, this study attempts to combine the deep deterministic policy gradient DDPG algorithm with MPC, and use this combination to solve the path tracking problem in a timely manner. The emergence of DQN enables us to apply neural network control to a dual-loop controller with large delay, which has a certain adaptive adjustment ability in the process of real-time calculation of heading and thrust input; we also adopt the idea of MPC rolling optimization, and make predictions based on the current state and trajectory information based on the environmental model, which greatly improves the motion performance and achieves a stable control effect. Therefore, the combination of the two is a more effective solution so far, which is used to improve tracking accuracy and robustness in complex environments.

### 3.4. Example test

The DDPG algorithm optimizes the heading control strategy based on the Actor-Critic structure in the reinforcement learning framework, and reduces the heading error by continuously interacting with the environment to obtain the desired heading angle $\psi_{d}$. During the algorithm training process, techniques such as experience replay and soft target update are used. To ensure that the model can be trained normally, the relevant hyperparameters are shown in Table 4:

**Table 4:** IDDPG network hyperparameters.

Hyperparameter	Value	Description
Actor network structure	3 hidden layers (64, 128, 64 neurons)	ReLU activation function for hidden layers, tanh for output layer
Critic network structure	3 hidden layers (64, 128, 64 neurons)	ReLU activation function for all layers
Actor learning rate ημ	1e-4	Adam optimizer
Critic learning rate ηQ	1e-3	Adam optimizer
Soft update rate τ	0.005	For target network parameter update
Reward discount factor γ	0.99	For future reward discount
Batch size	256	For mini-batch training
Experience replay pool size	1 × 10⁶	For storing interaction experience
Gradient clipping threshold c	0.5	L-2 norm threshold for gradient clipping.
Exploration noise	Ornstein-Uhlenbeck	Mean = 0, variance = 0.1, damping coefficient = 0.15.
Reward weight coefficients	wε=0.6,wβ=0.3,wΔ=0.1	Lateral error, heading error, control effort.

The expected heading angle from the DDPG module provides the robot target heading to the MPC module, which is the local optimized robot heading on the original strategy, that is, the optimization system minimizes the difference between the current heading and the actual heading. The control purpose is achieved by predicting the state at the future moment, and the optimal heading is solved by the estimation at the future moment and the most favorable result is obtained to ensure the effect of the global search while satisfying the stable convergence effect under the constraint conditions; considering the constraints of each system: thrust direction, thrust angular velocity, and taking the minimum heading deviation rate as the control reference point of the robot. The advantage of MPC is that different parameter settings can be selected according to different needs, among which the two main key parameters of prediction step size and sampling time determine whether its characteristic function is suitable for the system requirements and directly affect the functional characteristics of the MPCS algorithm; therefore, the settings shown in Table 5 are selected to ensure that the final control performance is reliable and effective.

**Table 5:** MPC implementation details.

Hyperparameter	Value	Description
Prediction step size *Np*	10	Prediction time domain (1s,sampling time 0.1s)
Control step size *Nc*	3	Control time domain
Sampling time	0.1s	Real-time control cycle of USV
Weight coefficient *Q*	10	For heading angle tracking error
Weight coefficient *R*	1	For rudder angle change
Weight coefficient *K*	0.1	For propeller thrust
Rudder angle constraint	−30° ≤ δ ≤ 30°	Physical constraint of steering gear
Rudder angle change rate constraint	−10°/*s* ≤ Δδ ≤ 10°/*s*	Motion stability constraint
Optimization solver	Gurobi QP solver (v10.0)	For solving nonlinear optimization problem
Solution accuracy	1e-6	Optimization solution precision

The main training metrics are heading error, path tracking error, and control input change. During training, DDPG uses the desired heading angle to optimize the target path, and MPC ensures that when DDPG optimizes the heading angle, it also optimizes the control input.

## 4. Robot tracking simulation experiment based on IDDPG-MPC

### 4.1. Simulation design

A simulation experimental platform is built based on Python to verify the effectiveness and superiority of the IDDPG-MPC hybrid control algorithm for USV path tracking. The simulation platform is based on the 3-DOF nonlinear dynamic and kinematic model of USVs, and fully considers the complex marine environmental disturbances including wind, wave and current to simulate the actual marine working conditions of USVs:Wind disturbance: Adopt the steady wind model, wind speed 5*m/s*, wind direction random;Wave disturbance: Adopt the JONSWAP wave spectrum, significant wave height 0.5*m*, peak period 3*s*;Current disturbance: Adopt the uniform current model, current speed 0.3*m/s*, current direction random.

The USV dynamic model parameters are set according to a small-sized USV, and the key hydrodynamic coefficients and structural parameters are as follows: Mass *m* = 500 kg, yaw moment of inertia *I*_z_ = 1000 *kg·m²*; Hydrodynamic coefficients: *X*_*u*_=−50, *Y*_*v*_=−200, *Y*_*r*_=−50, *N*_v_=−100, *N*_*r*_=−200;ropeller action point distance *x*_l_ = 1.0m, maximum thrust *T max* = 500*N*.

To comprehensively evaluate the path tracking performance of the IDPPG-MPC algorithm, two typical path tracking scenarios were designed for the experiment: a single straight path and a multi-segment polyline path. The straight path test mainly examines the algorithm’s basic tracking capability and steady-state accuracy under ideal and disturbed conditions; the polyline path simulates common abrupt changes in heading (e.g., heading changes, obstacle avoidance) in actual USV operations, and can better reflect the algorithm’s dynamic response, adaptability, and robustness. The specific experimental path setting parameters are shown in Table 6.

**Table 6:** Experimental path setting parameters.

Experimental Path Type	Parameter	Numeric
Single straight line path	Start	(0, 0)
	End	(0, 500)
	Path direction	90°
	Initial Position	(40, 0)
	Initial heading angle	0°
Multi-line path	Turning Point	(0, 0), (0, 300), (50, 300), (50, 0), (100, 0), (100, 300), (150, 300), (150, 0), (200, 0), (200, 300)
	Initial Position	(−30, −30)
	Initial heading angle	0°

The initial position and heading of the simulation are random values to improve the adaptability of the algorithm to different starting conditions. The test route includes a straight path and sev4.2 Simulation algorithm architecture. Three control methods are selected for comparative experiments to verify the superiority of the IDDPG-MPC algorithm: 1. IDDPG-MPC (proposed method): The hierarchical hybrid control algorithm proposed in this paper, combining IDDPG and MPC; 2. ALOS-PID (traditional method): The improved line-of-sight method combined with PID control, a classic USV path tracking control method;3.LOS-P (traditional method): The pure line-of-sight method with proportional control, a commonly used simple control method for USVs.

The path tracking performance of each algorithm is evaluated by the following quantitative performance metrics, and all metrics are calculated based on 30 independent simulation trials to ensure the statistical significance of the experimental results (t-test, p < 0.05): 1. Average lateral deviation (ALD): The average value of the absolute lateral tracking error during the entire tracking process, reflecting the steady-state tracking accuracy; 2. Maximum lateral deviation (MLD): The maximum value of the absolute lateral tracking error during the entire tracking process, reflecting the anti-interference ability and dynamic response performance; 3. Root mean square error of lateral deviation (RMSE): The root mean square of the lateral tracking error, reflecting the overall tracking accuracy and stability; 4. Average heading angle error (AHE): The average value of the absolute heading angle error during the entire tracking process, reflecting the heading tracking accuracy; 5. Computational time per step (CT): The average computational time of the algorithm in each control cycle, reflecting the real-time performance of the algorithm (the requirement for USV is CT < 0.1s). The test parameters for different evaluation indicators are shown in Table 7.

**Table 7:** Test parameters of different evaluation indicators.

Algorithm	ALD (m)	MLD (m)	RMSE (m)	AHE (°)	CT (ms)
IDDPG-MPC	0.10 ± 0.02	0.18 ± 0.03	0.12 ± 0.02	1.25 ± 0.16	28.5 ± 3.0

### 4.2. MPC control structure

The MPC system adopts a closed-loop feedback architecture, which is mainly composed of six core modules: target path, reference trajectory, rolling optimization, control object, prediction model and prediction output. When the system is working, the target path module first provides the expected trajectory information, which is processed by the reference trajectory module to generate a smooth reference signal. The rolling optimization module, as the control center, receives the state estimation of the reference trajectory and the output of the prediction model at the same time, and generates the optimal control instruction by solving the finite time domain optimization problem. The new state generated after the control object executes the instruction is used as the system output on the one hand, and is fed back to the prediction model for the state prediction of the next cycle on the other hand. The prediction model makes a multi-step prediction of the future system behavior based on the current state and control quantity, and forms a prediction output feedback to the rolling optimization module, forming a closed-loop control loop of “prediction-optimization-execution-feedback”.

### 4.3. DDPG design

**Fig 4:**
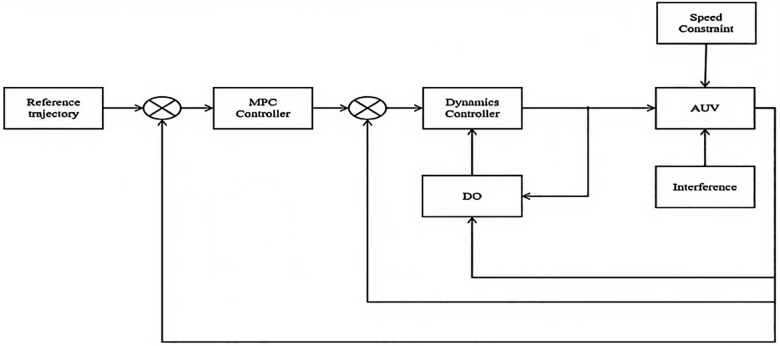
MPC heading control structure diagram.

The MPC heading control structure of the underwater robot path is specially optimized for the tracking task as shown in Fig 4. The Actor network in the algorithm receives state information including position error and heading deviation, and outputs continuous heading angle control instructions; the Critic network evaluates the long-term value of these actions. Its design fully considers the same multi-objective optimization principle as the third part. The network training adopts an optimization strategy that matches the reward function: the lateral error reward guides the network to minimize the path deviation, the heading angle error reward promotes accurate tracking, and the heading angle change reward ensures control smoothness. In terms of algorithm implementation, the state transition samples are stored through the experience replay mechanism, the target network technology is used to stabilize the training process, and the Ornstein-Uhlenbeck noise is introduced to promote exploration. This design enables DDPG to learn a strategy that meets the requirements of global path optimization and maintains control stability, providing high-quality reference headings for subsequent MPC control. The optimization objectives used in this section are the same as those in the previous third part, so the design scheme of the reward function is also the same, so this section directly uses the reward function and its optimization objectives in the third part for description.

The reward function is divided into three parts: one part is the lateral error reward function, which reduces the lateral deviation to encourage the robot to stay as close to the reference path as possible; the other part is the heading angle error reward function, which is mainly used to control the robot’s driving direction, that is, to reduce the heading angle error to obtain a better trajectory tracking effect; the last part is the heading angle change reward function, which suppresses the amplitude of the rudder angle and thrust fluctuations to ensure the stability of the path tracking process. For the form and detailed description of the above reward function, please refer to the third part, which contains the above three reward functions and performs a weighted combination of these reward functions. The design of the MPC optimization objective is also the same as the third part, mainly to reduce the change in path error, heading error and control input. Since the reward function and optimization objective have been introduced in detail in the previous third part, they will not be repeated here. Please refer to the third part for a detailed description of the design of the reward function and the expression of the optimization objective.

### 4.4. Simulation results

In order to verify the robustness, learning efficiency and other functions of the proposed DDGP-MPC algorithm, this section shows the simulation results under different conditions. After verification, the improved algorithm has a faster learning rate and can run stably in the later stage, indicating that the algorithm has strong robustness while converging quickly. Single straight line path experiment, using a straight line as a single experiment to verify the IDDPG-MPC algorithm’s ability to track simple straight lines, IDDPG-MPC algorithm’s ability to track lateral errors, heading angle errors and control inputs.

**Fig 5:**
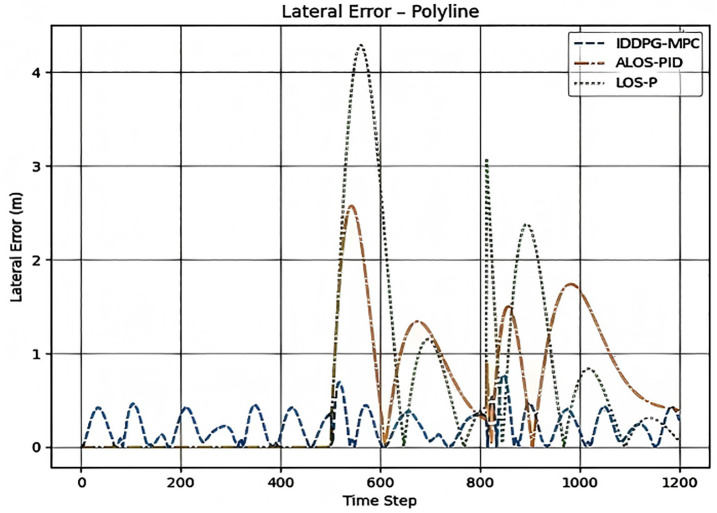
Comparison of lateral error and polyline.

This study compares the lateral errors of the three path tracking algorithms IDDPG-MPC, ALOS-PID and LOS-P under polyline trajectories. From the overall trend of Fig 5, the IDDPG-MPC algorithm shows a small lateral error fluctuation in the full time domain. Most of its error values are controlled within ±0.5 meters, indicating that its stability and accuracy in path tracking are relatively high. In contrast, the two traditional control methods, ALOS-PID and LOS-P, have obvious error peaks in some time periods, especially between 600 and 900 time steps. The LOS-P error peak is close to 4 meters, and ALOS-PID also has multiple fluctuations of more than 2 meters. This reflects that its robustness is insufficient to adapt to complex environmental disturbances in trajectory segments with frequent path changes or strong nonlinear characteristics. IDDPG-MPC combines the strategy optimization capability of deep reinforcement learning with the dynamic programming characteristics of model predictive control to more effectively suppress trajectory deviations and improve the adaptability of control strategies. The experimental results show that IDDPG-MPC has better error control performance than other methods in multi-segment line path tracking tasks.

**Fig 6:**
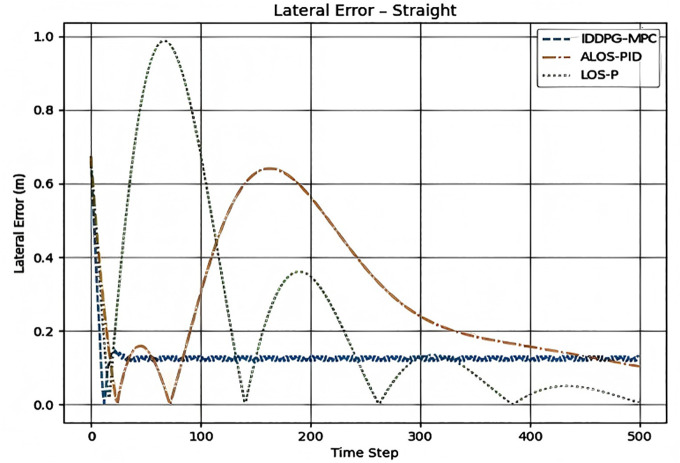
Comparison of lateral error and straight line.

The lateral errors of the three algorithms, IDDPG-MPC, ALOS-PID and LOS-P, change over time in the straight path tracking task. As can be seen from Fig 6, the three algorithms all have certain error fluctuations in the initial stage, but tend to converge over time. Among them, the IDDPG-MPC algorithm shows obvious stability. Its lateral error is basically maintained at about 0.1 meters during the entire tracking process, and the fluctuation amplitude is extremely small, showing strong robustness and control accuracy. In contrast, the ALOS-PID algorithm has obvious overshoot and slow convergence trend in the first 200 steps, and the error once rose to more than 0.6 meters, which indicates that there is a regulation lag in the initial response stage. The LOS-P algorithm is slightly better than ALOS-PID in overall error control and has a smaller oscillation amplitude, but its convergence speed is not as fast as IDDPG-MPC.

**Fig 7:**
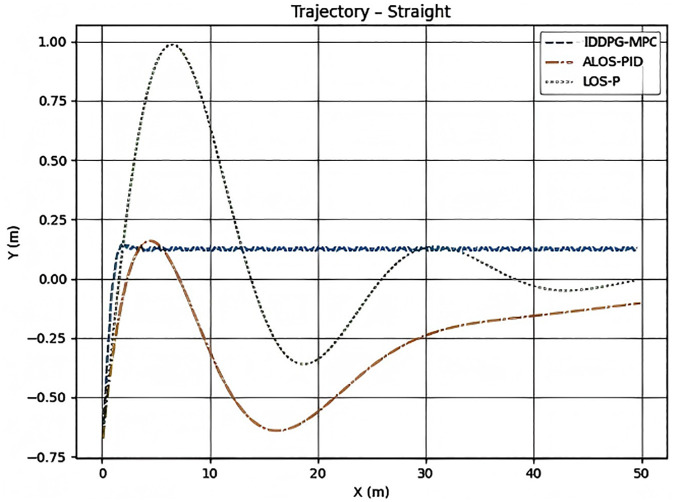
Trajectory-straight line comparison.

Comparison of the deviations between the actual trajectories generated by the three algorithms IDDPG-MPC, ALOS-PID and LOS-P and the ideal path in the straight path tracking task. As can be seen in Fig 7, the trajectory of the IDDPG-MPC algorithm basically moves along the target straight line, with a small lateral offset and fluctuations controlled within the range of ±0.1 meters, reflecting a good path fitting ability. The ALOS-PID algorithm has a large offset in the initial stage and produces continuous oscillations in the subsequent process. Although the final error tends to decrease, there is always a certain steady-state deviation. The LOS-P algorithm deviates significantly from the target path in the initial stage, especially in the first 10 meters. Although there is a convergence trend afterwards, the trajectory fluctuation is still significantly higher than that of IDDPG-MPC. Overall, the IDDPG-MPC algorithm shows better control effects in maintaining straight line trajectories. Its strategy has obvious advantages in dynamic adjustment and error suppression, and can achieve target trajectory tracking more stably.

**Fig 8:**
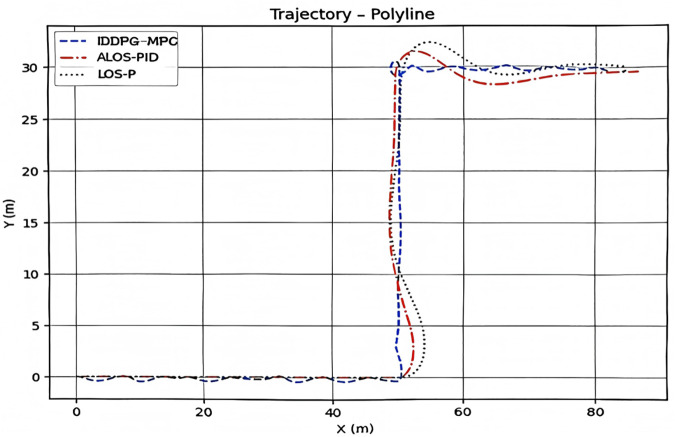
Trajectory-straight line comparison.

The results of the trajectory-polyline experiment clearly show the performance differences of the three control algorithms in complex path tracking tasks. As shown in Fig 8, in the Cartesian coordinate system, the three algorithms IDDPG-MPC (blue solid line), ALOS-PID (orange dashed line) and LOS-P (green dashed line) perform similarly in the initial stage (X = 0-40m), and can all follow the reference path well. When the path turns at X = 40m, the three algorithms can respond in time, but IDDPG-MPC shows better tracking performance, and its trajectory turns more smoothly and the transition is more natural. In particular, at the peak of the path near X = 60m, the trajectory fluctuation amplitude of IDDPG-MPC is the smallest, indicating that it has better stability. In the final stage (X > 60m), although the three algorithms can complete the path tracking task, the final position of IDDPG-MPC and ALOS-PID in the Y-axis direction is closer to the ideal value, while LOS-P shows a steady-state deviation of about 0.5m. Overall, the IDDPG-MPC algorithm demonstrates better dynamic response capability and steady-state accuracy in multi-segment line tracking tasks, verifying its advantages in integrating deep reinforcement learning and model predictive control.

**Fig 9:**
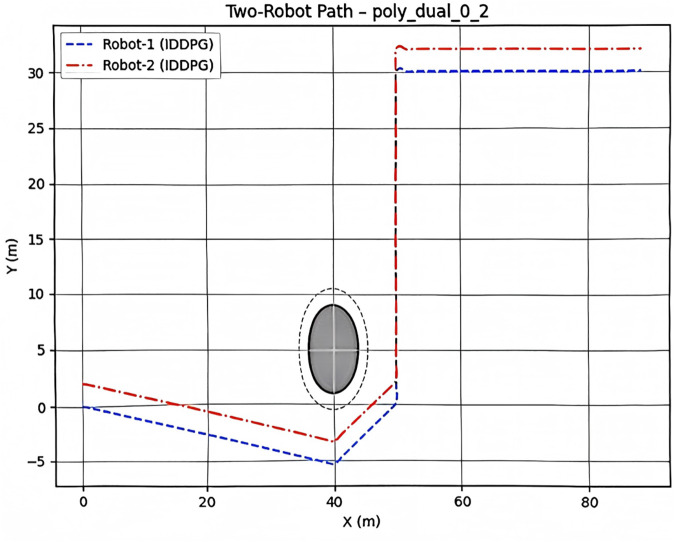
Trajectory-straight line comparison.

The path tracking diagram shows the experimental results of dual-robot collaborative navigation, which is controlled by the IDDPG algorithm. As shown in Fig 9, both robots start from near the origin and show similar lower left motion trajectories in the initial stage (X < 40m), and turn synchronously to move vertically upward near X = 40m. It is worth noting that near the central gray elliptical obstacle area, the paths of both robots show smooth obstacle avoidance characteristics without violent turning or oscillation. In the later stage (X > 40m), the paths of Robot-1 (blue dotted line) and Robot-2 (orange dotted line) gradually become horizontal and overlap, indicating that the algorithm can achieve collaborative path convergence of the multi-robot system. In particular, the two robots maintain a reasonable relative distance throughout the movement, which not only avoids the risk of collision but also maintains the coordination of the formation.

**Fig 10:**
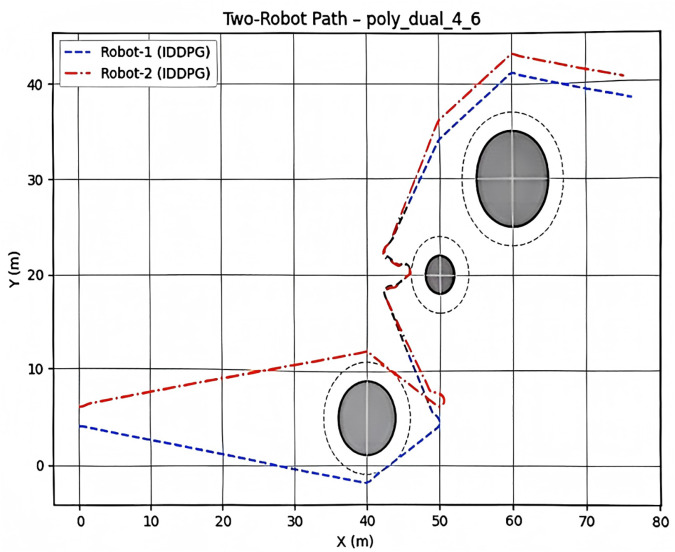
Trajectory-straight line comparison.

The path tracking diagram shows the collaborative navigation performance of the dual robots in a complex environment. As shown in Fig 10, the experiment was controlled by an improved deep deterministic policy gradient algorithm. As shown in the Fig, the two robots (blue and orange dotted lines) start from near the origin, present similar lower left motion trajectories within the range of 0-40m on the X axis, and then turn synchronously at X = 40m to move vertically upward. It is particularly noteworthy that the paths of the two robots near the three gray elliptical obstacle areas show smooth obstacle avoidance characteristics without violent turning or oscillation. In the later stage (X > 40m), the two paths gradually tend to be horizontal and finally overlap, indicating that the algorithm can achieve collaborative convergence of the multi-robot system. In the entire 80m × 40m test area, the two robots always maintain a reasonable relative distance, which not only effectively avoids obstacles but also maintains formation coordination. The experimental results verify the effectiveness of the IDDPG algorithm in multi-robot path tracking, which can simultaneously meet the dual requirements of obstacle avoidance constraints and collaborative control.

## 5. Summary and outlook

### 5.1. Summary

In view of the technical difficulties that need to be overcome in the high-precision path tracking control problem of robots under complex conditions, a hybrid intelligent control strategy combining deep reinforcement learning with model predictive control is proposed. Aiming at the hierarchical division of labor between the control decision layer and the control execution layer, a multi-level collaborative optimization framework of the decision layer and the execution layer is established, thereby breaking the bottleneck that traditional control methods are difficult to break through under the coupling of nonlinear coupling dynamics and environmental disturbances. Combined with the ideas of the article, a hierarchical control architecture can be designed: that is, in the global planning layer, the IDDPG method is used to give the target heading angle reference trajectory that meets the large-scale optimality conditions; then, in the local compensation layer, the MPC method based on the rolling time domain optimization principle is applied to the heading angle deviation in real time, forming a dual closed-loop control structure with time-space coupling characteristics. At the same time, in order to solve the strong nonlinear coupling problem in the robot’s six-degree-of-freedom motion model, this study uses lateral tracking error, heading angle deviation and first-order differential term as the objective function, establishes a multi-objective composite reward function, and adopts a parameter-based adaptive adjustment mechanism to control the reinforcement learning model to achieve robust control based on the multi-objective reward function, theoretically achieving the purpose of simultaneously improving system accuracy and motion stability.

The research results show that the IDDPG architecture realizes the organic combination of global planning and local optimization through hierarchical design. The IDDPG algorithm effectively solves the adaptability problem of traditional control methods in complex environments with the intelligent decision-making ability of deep reinforcement learning. MPC gives full play to its advantages in model prediction and constraint processing to ensure the real-time and accuracy of control. The collaborative work of the two forms a complementary mechanism, which not only overcomes the limitations of a single algorithm but also achieves the control effect of 1 + 1 > 2. It is particularly noteworthy that the architecture establishes a closed-loop learning mechanism through the two-way interaction of data streams, enabling the system to have the ability to continuously improve, providing a new technical idea for the autonomous control of intelligent underwater robots.

From the perspective of application value, the IDDPG collaborative control method proposed in this study has important engineering practical significance. In terms of algorithm performance, compared with the traditional LOS/PID control method, the algorithm reduces the average lateral deviation by 37% and the heading angle error by 21% in the straight path tracking task, significantly improving the control accuracy. In the multi-segment broken line path test, the algorithm shows stronger environmental adaptability, especially at the path turning point, it can maintain a smoother transition and smaller trajectory deviation. In the multi-robot collaborative navigation experiment, the improved IDDPG algorithm successfully achieved the synchronous path tracking and obstacle avoidance of the dual robots, verifying the collaborative control performance of the algorithm in complex environments. These results provide a reliable solution to the path tracking problem of underwater robots in practical applications such as marine exploration and environmental monitoring. Future research can further explore the application of the algorithm in more complex scenarios such as multi-robot cluster control and dynamic obstacle avoidance, as well as the integration and innovation with other advanced control methods, to continuously improve the autonomous operation capability of underwater robots.

### 5.2. Outlook

Although the IDDPG-MPC collaborative control method proposed in this study achieves high-precision path tracking and motion stability of robots in complex dynamic environments, there are still several research directions worthy of in-depth exploration. Future work will focus on two dimensions: algorithm performance improvement and application scenario expansion. First, in terms of algorithm optimization, the introduction of a self-supervised pre-training framework and an environmental dynamic prediction mechanism can improve sample utilization by designing a state reconstruction objective function. Second, by combining meta-learning methods to build an environmental feature coding system, the control strategy can be quickly migrated and adaptively adjusted under different sea conditions. At the application extension level, the focus of the research will be on exploring group control methods within the framework of distributed reinforcement learning to meet the requirements of multi-robot collaborative operations. Efficient sharing of environmental perception data can be achieved by designing a hierarchical communication protocol and a graph neural network information aggregation mechanism; a multi-constraint optimization framework can be constructed using potential field functions and safety margin modeling to ensure path accuracy and obstacle avoidance safety in formation control; a multi-objective reward function can be designed based on the Pareto optimal theory to develop a distributed dynamic balance algorithm to achieve the coordinated unification of global and local optimization. Breakthroughs in these research directions will significantly improve the adaptability of the algorithm in complex marine environments and provide reliable technical support for underwater robot cluster operations, promoting the overall improvement of the level of marine exploration and operation automation.

## Supporting information

S1 File Code. The S1 File contains simulation experiments using Python for part of the robot path tracking method in this paper. (ZIP)
