# Supplementary figures and images for: Research on robot path tracking method based on IDDPG-MPC
Source: PLoS One. 2026 Jul 6;21(7):e0350307. doi: 10.1371/journal.pone.0350307 (PMC13336202; doi:10.1371/journal.pone.0350307)

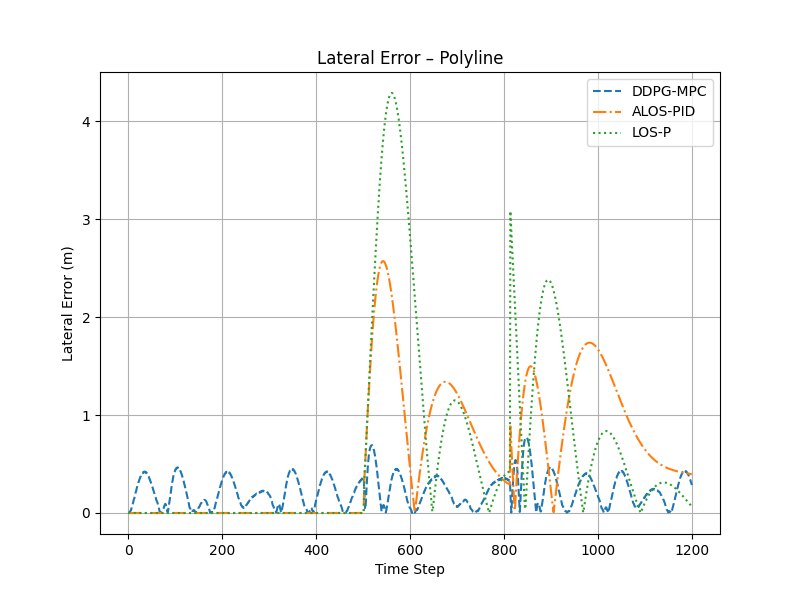

Supplement: S1 File — (ZIP) [file pone.0350307.s001.zip › code/code/UsvPathFollowing/results/lateral_error_polyline.png]

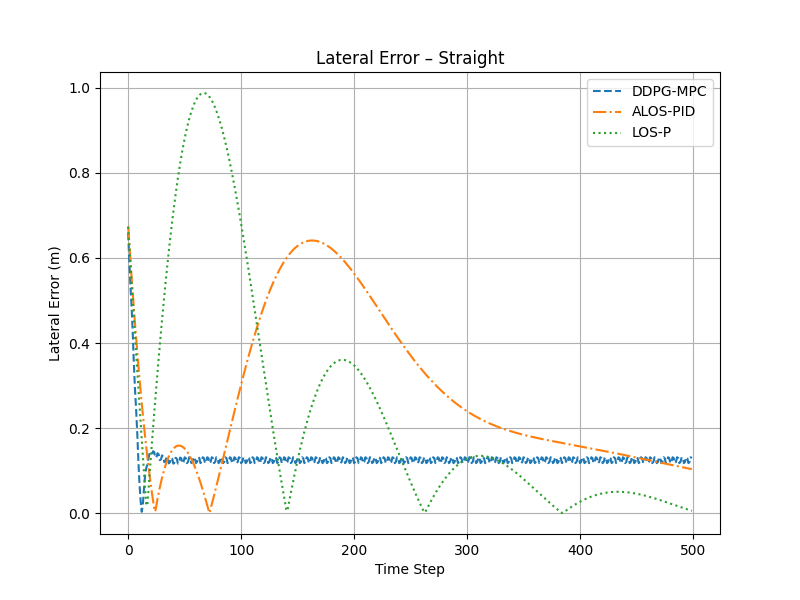

Supplement: S1 File — (ZIP) [file pone.0350307.s001.zip › code/code/UsvPathFollowing/results/lateral_error_straight.png]

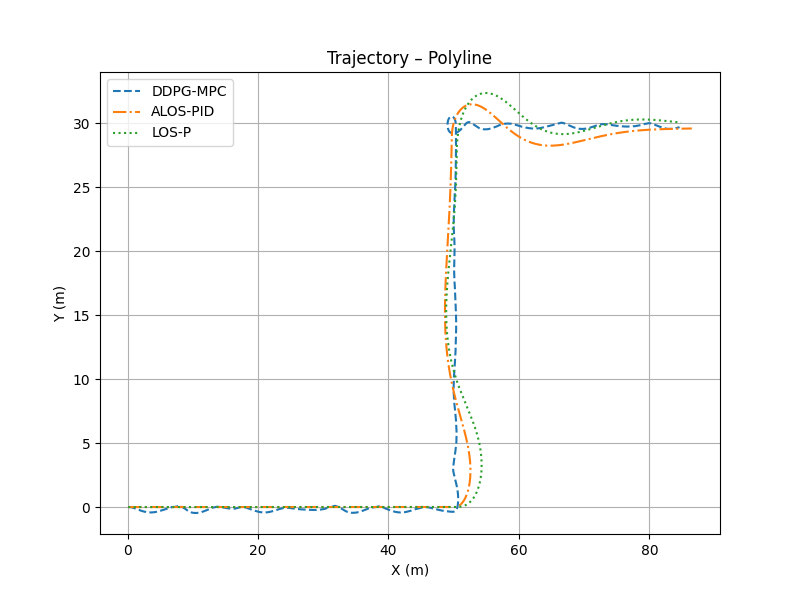

Supplement: S1 File — (ZIP) [file pone.0350307.s001.zip › code/code/UsvPathFollowing/results/trajectory_polyline.png]

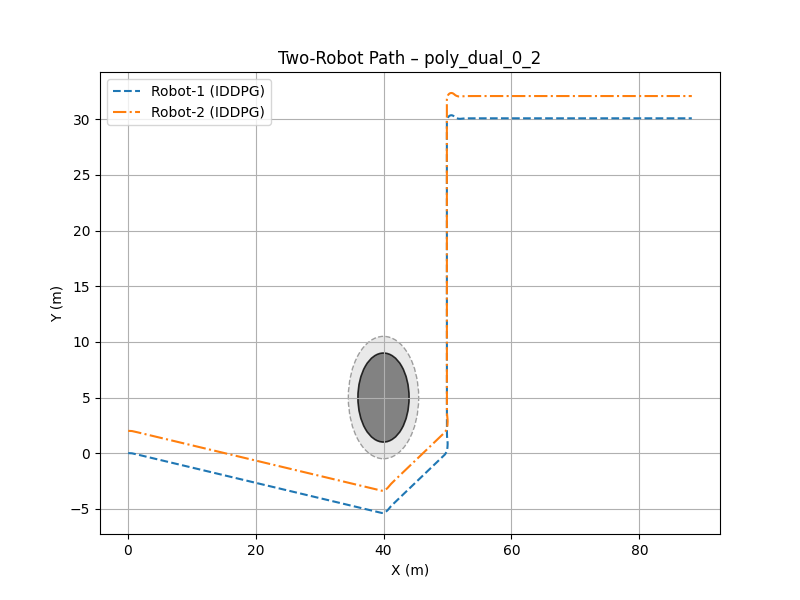

Supplement: S1 File — (ZIP) [file pone.0350307.s001.zip › code/code/UsvPathFollowing/results/trajectory_poly_dual_0_2.png]

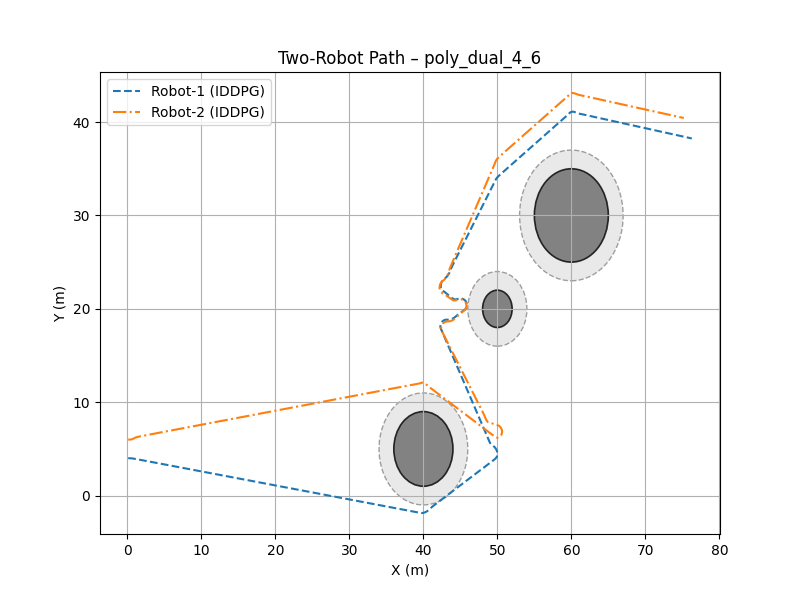

Supplement: S1 File — (ZIP) [file pone.0350307.s001.zip › code/code/UsvPathFollowing/results/trajectory_poly_dual_4_6.png]

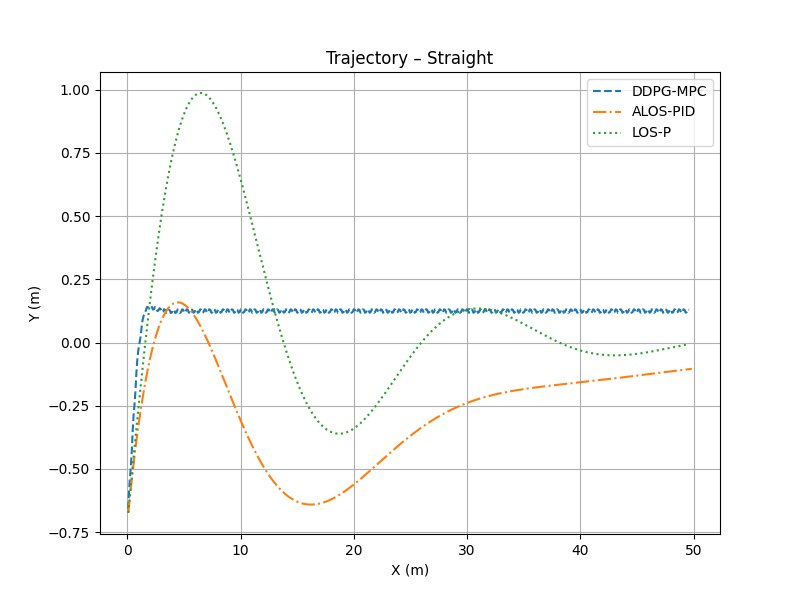

Supplement: S1 File — (ZIP) [file pone.0350307.s001.zip › code/code/UsvPathFollowing/results/trajectory_straight.png]
